# Schistosome Interactions within the *Schistosoma haematobium* Group, Malawi

**DOI:** 10.3201/eid2506.190020

**Published:** 2019-06

**Authors:** Bonnie L. Webster, Mohammad H. Alharbi, Sekeleghe Kayuni, Peter Makaula, Fenella Halstead, Rosie Christiansen, Lazarus Juziwelo, Michelle C. Stanton, E. James LaCourse, David Rollinson, Khumbo Kalua, J. Russell Stothard

**Affiliations:** Natural History Museum, London, UK (B.L. Webster, D. Rollinson);; Ministry of Health, Qassim, Saudi Arabia (M.H. Alharbi);; Liverpool School of Tropical Medicine, Liverpool, UK (M.H. Alharbi, S. Kayuni, F. Halstead, R. Christiansen, E.J. LaCourse, J.R. Stothard);; Medical Aid Society of Malawi, Blantyre, Malawi (S. Kayuni);; Research for Health Environment and Development, Mangochi, Malawi (P. Makaula);; Ministry of Health, Lilongwe, Malawi (L. Juziwelo);; Lancaster University Medical School, Lancaster, UK (M.C. Stanton);; Lions Sight First Eye Hospital, Blantyre (K. Kalua)

**Keywords:** hybridization, introgression, co-infection, evolution, zoonotic potential, *Schistosoma haematobium*, zoonoses, Malawi, parasites, trematodes

## Abstract

Molecular analysis of atypical schistosome eggs retrieved from children in Malawi revealed genetic interactions occurring between human (*Schistosoma haematobium*) and livestock (*S. mattheei* and *S. bovis*) schistosome species. Detection of hybrid schistosomes adds a notable new perspective to the epidemiology and control of urogenital schistosomiasis in central Africa.

Urogenital schistosomiasis is a waterborne disease transmitted by certain freshwater snails that occurs throughout much of sub-Saharan Africa. Until recently, this disease was attributed solely to *Schistosoma haematobium*, which was considered to have limited zoonotic potential (*1*). However, genetic analysis of natural infections with noninvasive larval sampling (*2*) has provided new evidence. In West Africa, for example, species interactions with hybrid combinations of *S. haematobium* and the bovine or ovine species of *S. bovis* and *S. curassoni* are commonly encountered in humans and snails (*3*). Although key biologic features of hybrids may not always be apparent, the risk for zoonotic transmission along with enhanced definitive and intermediate host compatibilities needs investigation (*2*,*3*). The recent emergence and persistent transmission of *S. haematobium*–*bovis* hybrids on the Mediterranean island of Corsica (*4*) demonstrates the public health impact of such genetic introgression.

Genetic analysis of *S. haematobium* group species in central and southern Africa is a high priority. Atypical egg morphologies suggest a capacity for natural hybridization of *S. haematobium* with the bovine species *S. mattheei*, later confirmed with biochemical markers and experimental infections demonstrating viable progeny (*3*). During ongoing surveillance of urogenital schistosomiasis in Chikhwawa District, Malawi, we encountered atypical *S. haematobium* eggs in urine samples from several infected children (*5*). We report the further genetic characterization of atypical eggs collected from epidemiologic surveys of children within Chikhwawa, Nsanje, and Mangochi Districts (Figure, panel A). 

**Figure:**
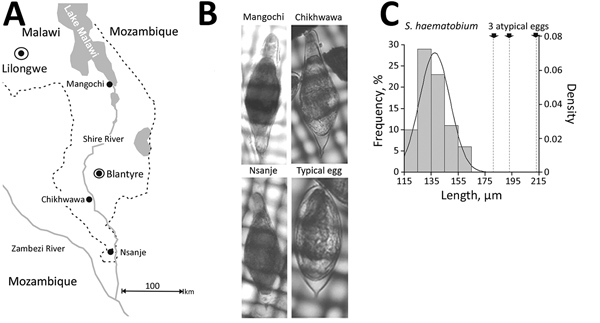
Investigation of atypical schistosome eggs retrieved from children in Malawi. A) Locations where urine samples containing *Schistosoma haematobium* eggs were collected from children in Mangochi (Samama village, 14°41′74.65′′S, 35°21′75.80′′E), Chikhwawa (Mpangani village, 16°03′62.99′′S, 34°84′10.63′′E), and Nsanje (Kastiano village, 16°90′63.98′′S, 35°26′65.78′′E) districts. Of the children sampled, ≈10% had atypical eggs in their urine, in an approximate atypical:typical ratio of 1:25. Note that the Shire River flows southward from Lake Malawi, linking the 3 sampled locations within the same drainage basin. B) Photomicrographs of a representative atypical egg from each location. Corresponding genotypes assigned for the mitochondrial *cox*1 and nuclear rITS loci: Mangochi, *cox*1 *S. mattheei* and rITS *S. haematobium-mattheei*; Chikhwawa, *cox*1 and rITS *S. haematobium*; Nsanje, *cox*1 *S. bovis* and rITS *S. haematobium*. A typical *S. haematobium* egg is shown for comparison. Sizes are not too scale. C) Histogram of length measurements for 83 typical *S. haematobium* eggs collected from Nsanje. Solid line indicates the associated density distribution. The mean length of this sample of typical eggs was 135 ± 28 µm (1 SD), with minimum 86 µm and maximum 180 µm. Arrows with dashed lines at right indicate the length of the 3 atypical eggs, which fall well outside the range of length variation of the 83 typical eggs as measured.

Ethics approvals for the epidemiological surveys were granted by Liverpool School of Tropical Medicine; College of Medicine, Malawi; and Ministry of Health and Population, Malawi. All children found infected were treated with praziquantel.

We filtered schistosome eggs from the urine of infected children, then photographed and measured them before storing them on Whatman FTA cards for molecular analysis (*2*). We alkaline-eluted and genotyped DNA from individual eggs using both the mitochondrial cytochrome oxidase subunit 1 (*cox*1) and the nuclear ribosomal internal transcribed spacer (rITS) DNA regions (*2*) (Appendix Table). In addition, for the samples from Mangochi District, we analyzed a partial region (300-bp) of the nuclear ribosomal 18S DNA to confirm the presence of *S. mattheei* nuclear DNA (*2*,*6*) (Appendix). 

Of 6 atypical eggs from Chikhwawa, all had a pure *S. haematobium* genetic profile (Figure, panels B, C). Of 19 eggs from Nsanje, 18 had a pure *S. haematobium* genetic profile; 4 eggs had atypical morphology, but only 1 atypical egg had a discordant genetic profile (i.e., *cox*1 *S. bovis* and rITS *S. haematobium*). Of 20 eggs from Mangochi, 16 typical *S. haematobium* eggs had a pure *S. haematobium* genetic profile, whereas the 4 atypical eggs had the same discordant genetic profiles (*cox*1 *S. mattheei* and rITS *S. haematobium-mattheei*). Inspection of the partial 18S gene sequence confirmed *S. haematobium–mattheei* hybrids (Appendix). We deposited all sequence data into GenBank (accession nos. MK358841–MK358858).

Our genetic analysis demonstrated the presence of *S. haematobium* group hybrids in Malawi as introgressed forms of *S. haematobium–mattheei* and *S. haematobium–bovis*. Of note, an unusual egg morphology may not always correspond with the ability to detect introgression with the current combination of genetic markers used (*6*; Appendix). As described by Boon et al., successive backcrossings of hybrid progeny may obscure our ability to detect ancestral introgression, and the development of a wider panel of nuclear genetic markers is needed (*6*). Nonetheless, detection of these 2 hybrid schistosomes strongly suggests interactions of *S. haematobium* with the ungulate schistosomes *S. mattheei* and *S. bovis*. That *S. bovis* has not been reported in Malawi implies a changing species dynamic with possible zoonotic transmission along the drainage basin of Lake Malawi, adding a new dimension to the epidemiology and control of urogenital schistosomiasis in Malawi (*7*).

Because we did not attempt miracidial hatching during this study, we cannot confirm that these hybrids or introgressed forms are fully viable in autochthonous natural transmission. However, the process of ancestral introgression with subsequent natural selection may help explain unexpected shifts in local snail–schistosome relationships (e.g., the changing compatibility of *Bulinus nyassanus* snails in Lake Malawi with *S. haematobium* schistosomes) (*8*). Further studies are needed to better characterize schistosomes involved in human infection, investigate more thoroughly any zoonotic potential, and assess all possible combinations of interspecies introgressions. 

Molecular evidence for ancestral hybridization between *S. haematobium* and *S. mansoni* schistosomes was presented recently (*9*); given autochthonous transmission of intestinal schistosomiasis in Lake Malawi (*10*), there may be sufficient epidemiologic opportunity for other introgression events to occur with the hybrids we report. We therefore advise heightened concurrent surveillance of urogenital and intestinal schistosomiasis, entailing a OneHealth approach with molecular vigilance for interspecies interactions along with phenotypic assessments for any altered host pathogenicity or susceptibility to praziquantel treatment. Detection of the hybrid schistosomes we report adds a new perspective to the epidemiology and control of urogenital schistosomiasis in central Africa.

Appendix Additional information about *Schistosoma haematobium* hybridization, Malawi.
