# Supplementary material for: Schistosome Interactions within the Schistosoma haematobium Group, Malawi
Source: Emerg Infect Dis. 2019 Jun;25(6):1245–7. doi: 10.3201/eid2506.190020 (PMC6537718; doi:10.3201/eid2506.190020)
Supplement: Appendix — Additional information about Schistosoma haematobium hybridization, Malawi. [file 19-0020-Techapp-s1.pdf]

Article DOI: https://doi.org/10.3201/eid2506.190020

Schistosome Interactions within the *Schistosoma haematobium* Group, Malawi

Appendix

DNA Extraction

DNA preparation from FTA stored schistosome eggs and larvae:

1. Add 14 µL of Solution 1 (0.1M NaOH, 0.3mM EDTA, pH13.0) to the punchout 2 mm FTA disc.
2. Incubate at room temperature for 5 min.
3. Add 26 µL of Solution 2 (0.1M Tris-HCl, pH7.0).
4. Pulse vortex 3 times.
5. Incubate at room temperature for 10 min.
6. Pulse vortex 10 times.
7. Use 3 µL of the DNA elution in a 25 µL PCR.

PCR Analysis and Sequencing

We eluted DNA as described above from the schistosome eggs stored on the FTA cards. In separate PCRs, run on a Perkin Elmer 9600 Thermal Cycler, we amplified the *cox*1, ITS and 18S DNA regions (Appendix Table). We performed a 25 µL PCR reaction for each using illustraTM puReTaq Ready-To-Go PCR Beads (GE Healthcare, Hertfordshire, UK, https://www.gehealthcare.com) and 10 pmol of each primer (Appendix Table) and 3µL of the DNA elution.

We checked all PCR reactions for positive amplification of the correct band size by gel electrophoresis using 0.8% Gelred agarose gels (Biotium, https://biotium.com). We purified PCR amplicons and Sanger sequenced them in both directions using a dilution of original PCR primer.

We used Sequencher version 5.1 (Gene Codes Corp., http://www.genecodes.com) to visualize and manually edit all sequence data.

We confirmed mitochondrial *cox*1 sequence identity using the Basic Local Alignment Search Tool (BLAST) (https://blast.ncbi.nlm.nih.gov/Blast.cgi). We analyzed the ITS and 18S sequence identity by visual comparison to personal reference sequences for each species (*S. haematobium*, *S. bovis*, and *S. mattheei*). We visually checked known interspecies SNP regions (Appendix Figure) to identify homogenous or heterogenous ITS and 18S DNA.

We inspected mitochondrial and nuclear genetic profiles to identify hybrids (and any discordance of mitochondrial and nuclear DNA data).

The mean egg length of this sample of 83 typical eggs was 135 ± 28µm (1 SD), which was very similar to the 137 ± 15µm (1 SD) reported by Boon et al (*1*). We referred to additional sources on unusual egg morphology (*2*–*4*).

References

1. Boon NAM, Fannes W, Rombouts S, Polman K, Volckaert FAM, Huyse T. Detecting hybridization in African schistosome species: does egg morphology complement molecular species identification? Parasitology. 2017;144:954–64. PubMed http://dx.doi.org/10.1017/S0031182017000087

2. Blackie WK. A helminthological survey of Southern Rhodesia. Memoir (London School of Hygiene and Tropical Medicine), no. 5. 1932, p. 1–91.

3. Pitchford RJ. Differences in the egg morphology and certain biological characteristics of some African and Middle Eastern schistosomes, genus *Schistosoma*, with terminal-spined eggs. Bull World Health Organ. 1965;32:105–20. PubMed

4. Sapp SGH, Yabsley MJ, Bradbury RS. Abnormal helminth egg development, strange morphology, and the identification of intestinal helminth infections. Emerg Infect Dis. 2018;24:1407–11. PubMed http://dx.doi.org/10.3201/eid2408.180560

Appendix Table. PCR primers used to amplify each DNA region and their associated PCR thermal cycle in study of *Schistosoma haematobium* hybridization, Malawi

DNA region	Forward primer (name)	Reverse primer (name)	PCR thermal cycle
ITS1+2 rDNA	TGCTTAAGTTCAGCGGGT (ITS1)	AACAAGGTTTCCGTAGGTGAA (ITS2)	5 min at 95°C:<br>40 cycles of 30 s at 95°C, 30 s at 58°C, 1.30 min at 72°C:<br>10 min at 72°C.
Partial 18S rDNA	GCGAATGGCTCATTAAATCAG (WA)	TCCGGAGAGGGAGCCTGA (300R)	5 min at 95°C:<br>40 cycles of 30 s at 95°C, 30 s at 60°C, 1 min at 72°C:<br>10 min at 72°C.
Partial *cox*1 mt DNA	TAATGCATMGGAAAAAAACA (cox1Schisto5′)	TCTTTRGATCATAAGCG (cox1Schisto3′)	5 min at 95°C:<br>40 cycles of 30 s at 95°C, 30 s at 40°C and 1.30 min at 72°C<br>10 min at 72°C.

Marker	18S (300bp)			ITS (906bp)										
SNP	138	163	210	18	26	50	51	91	92	120	170	225	490	877
S. haematobium	T	C	T	C	T	G	T	C	G	C	G	C	T	T
S. bovis	C	T	C	C	T	A	T	C	G	T	A	T	T	C
S. mattheei	C	T	T	T	A	A	C	T	A	T	A	T	A	T

Appendix Figure. Comparison of DNA sequences from eggs of 3 *Schistosoma* species, Malawi.
